# The Cross-Cultural Adaptation and Validation of the Persian Version of Dental Discomfort Questionnaire (P-DDQ)

**DOI:** 10.30476/DENTJODS.2021.91652.1596

**Published:** 2022-12

**Authors:** Arghavan Behbahanirad, Mohammadjavad Yousefimaaghoul, Mehrdad Vossoughi

**Affiliations:** 1 Dept. of Dental Public Health, School of Dentistry, Shiraz University of Medical Sciences, Shiraz, Iran; 2 Undergraduate Student, Student Research Committee, School of Dentistry, Shiraz University of Medical Sciences, Shiraz, Iran; 3 Oral and Dental Disease Research Center, Dept. of Dental Public Health, School of Dentistry, Shiraz University of Medical Sciences, Shiraz, Iran

**Keywords:** Pain, Child, Toothache, Dentistry, Validation study

## Abstract

**Statement of the Problem:**

It seems essential to detect toothache in children through a validated instrument. Dental Discomfort Questionnaire (DDQ) could specifically detect the behaviors that indicate toothache through parental reports.

**Purpose:**

Current study aimed to conduct a cross-cultural adaptation process and test the validity and reliability of the Persian version of DDQ (P-DDQ).

**Materials and Method:**

In this cross sectional study, 60 children aged 25 to 60 months and their parents who referred to the Pediatric Department of Shiraz Dental School were selected. Parents filled out the DDQ and one examiner recorded the dmft index. To adapt the DDQ questionnaire, conceptual and item equivalence, semantic equivalence and operational equivalence were performed. The dimensions of P-DDQ were evaluated using exploratory factor analysis (EFA). Test-retest reliability and internal consistency assessments were respectively carried out using weighted kappa coefficient (kw) and Cronbach’s alpha. Moreover, the construct validity was evaluated through Mann-Whitney U test and Spearman’s correlation coefficient. Kruskal-Wallis H and Dunn’s post-hoc tests were applied for discriminant validity.

**Results:**

The cross-cultural adaptation of P-DDQ was conducted and minor necessary modifications were applied. Test-retest reliability showed moderate and high levels of agreement for DDQ items except for two items. An acceptable internal consistency was observed between DDQ items (Cronbach’s alpha= 0.769). EFA showed that almost all 12 items of the questionnaire were included in three factors. Both construct and discriminant validities were established for P-DDQ.

**Conclusion:**

P-DDQ was cross-culturally well-adapted, validated, and reliable questionnaire applied with the purpose of detecting toothache among children in Iran.

## Introduction

The disability of caregivers to recognize pain in young children has always been considered as a challenge [ 1 - 2 ]. Due to the fact that pain is a subjective and multidimensional issue, its recognition varies among individuals and the only standard tool for pain assessment is the self-report of individuals [ 1 ]. Moreover, it has more importance among young children because they are not cognitively developed enough to remember or report the pain [ 2 ].

Dental pain as the consequence of dental caries is a common pain during the childhood [ 3 ]. It should be detected as soon as possible to prevent further clinical, psychological, and functional consequences [ 4 - 5 ]. However, most of the parents and non-dental health care workers are not able to recognize the dental pain at early stages of dental caries in young children. Therefore, validated behavioral dental pain assessment tools have to be applied in order to detect toothache in young children [ 6 ]. Dental Discomfort Questionnaire (DDQ) can specifically detect the behaviors indicative of dental pain through parental reports of 2-5 year-old children [ 7 ]. Afterwards, preventive and restorative procedures can be implemented before more severe consequences [ 8 ]. 

The English version of DDQ, which is the original one, was developed by Versloot et al. in Netherlands [ 7 ] and then, it was assessed by several studies [ 9 - 12 ]. They have shown that DDQ could be applied as an appropriate tool for the early prediction of toothache and dental caries in preverbal children. Furthermore, it was applied for children with learning disabilities or autism, and it was found that DDQ implementation for these children was highly beneficial [ 13 - 15 ]. Another study revealed that DDQ was a sensitive tool before and after performing the dental treatments for children [ 16 ]. 

The pain-related behaviors of children could be affected by the cultural context of communities; therefore, DDQ has to be cross-culturally adapted prior to its implementation in various cultures. The Brazilian version of DDQ has been developed and cross culturally adapted [ 11 - 18 ]. They revealed the final Brazilian-Portuguese version of DDQ for the Brazilian culture [ 11 ]. 

To the best of our knowledge, there are not any validated and comprehensive dental pain assessment tools for young children in Iran. In a study in Iran, this questionnaire was applied without considering standard validity and reliability steps [ 19 ]. Moreover, it seems essential to detect the dental pain and discomfort in young children using a validated and culturally adapted tool due to the high prevalence of dental caries in young children [ 20 ] Current study aimed to conduct a cross-cultural adaptation process and assess the validity and reliability of the Persian version of the DDQ (P-DDQ) for the first time.

## Materials and Method

This cross-sectional study was conducted with the approval of the Ethics Committee of Shiraz University of Medical Sciences (IR.Sums.Dental.REC.1399.046). According to the reported correlation coefficient between DDQ score and dmft (r= 0.40) [ 10 ] as well as values of α = 0.05 and β= 0.2, the sample size was determined to be 47. To increase the accuracy, 60 children aged 25 to 60 months and their parents were included in the study. This sample was chosen from the patients who referred to the Pediatric Department of Shiraz Dental School. The informed consent of all parents was achieved; children who suffered from mental or physical disabilities, or were in mixed dentition stage or did not have complete primary dentition or enough cooperation were excluded from the study.

DDQ, which included two main sections, had to be filled out by parents. The first section evaluated the toothache experience of children, while the second one assessed their toothache-related behaviors and oral habits. The 3-point Likert scale (0= never, 1= sometimes and 2= often) were specified for each item.

One calibrated examiner recorded the oral health of children, using dmft index in accordance with World Health Organization criteria [ 13 ]. Children were examined sitting on a chair using a head light, and disposable dental mirror and probe.

The cross-cultural adaptation process that included the conceptual and item equivalence, semantic equivalence, and operational equivalence was conducted [ 12 - 14 ] with the purpose of adapting DDQ to Iranian population. For conceptual equivalence, literature review implemented to compare the dental pain and discomfort concept in Iranian context and the original DDQ. Then an expert panel was designed with the purpose of assessing the conceptual and item equivalence. The panel was composed of two Iranian dental public health specialists and two Iranian pediatric dentists who appraised the concepts of dental pain in children and all 12 items of the English version of DDQ. To perform item equivalence, the panel members evaluated the relevancy of each item in Iranian context by choosing one of the 4-point Likert scale including relevant; relevant, but needing minor modifications; little relevance; and not relevant.

The purpose of applying the semantic equivalence was to translate-back translate the English DDQ into Persian; therefore, there were similar concepts between the original version and the Persian one [ 16 - 17 ]. Semantic equivalence included four steps: first of all, the translation of the DDQ into Persian by two native Iranian translators. One of these translators was a pediatric dentist who was fluent in English, and the other one was a professional English-to-Persian translator. Then, the back translation process was conducted by two native English speaker translators. Afterwards, an expert panel that was consisted of two Iranian dental public health specialist, two Iranian pediatric dentists, and two translators compared the translated and back-translated versions and produced a consensus Persian version. Finally, the back-translation of the consensus version was sent to the developer of the original DDQ to be verified.

Operational equivalence was performed to preliminarily test the instrument in the target population and modify the items according their concepts. This stage led to the development of a cross-culturally adapted version.

### Exploratory factor analysis and internal consistency

P-DDQ dimensions were evaluated by exploratory factor analysis (EFA). For this purpose, orthogonal varimax rotation was applied; also, the Bartlett test and Kaiser–Meyer–Olkin (KMO) were implemented in order to test the assumption of sphericity and measure the sampling adequacy, respectively. Items with factor loading values greater than 0.45 were considered as effective items in the relevant factor [ 15 ].

To assess the test-retest reliability, weighted kappa coefficients (kw) of 20 participants were computed two weeks after the completion of the first questionnaire. Moreover, Cronbach’s alpha was applied to test the internal consistency of the adapted version of DDQ.

### Construct and discriminant validity

To assess the construct validity, participants were categorized into two groups, based on toothache experience. Then, the total DDQ scores of the both groups were compared using Mann-Whitney U test; also, the correlation between DDQ score and the number of decayed teeth and dmft was examined by Spearman’s correlation coefficient.

To assess the discriminant validity, patients were divided into three clinical groups including children with toothache and decayed teeth (group 1, n=48), children without toothache but with decayed teeth (group 2, n=6) and children without toothache or decayed tooth (group 3, n=6). The total score of DDQ was then compared between the mentioned three groups using Kruskal-Wallis H and Dunn’s post-hoc tests.

### Statistical software used to analyze data

All of the statistical analysis was performed using IBM SPSS Statistics for Windows, version 22.0 (IBM Corp, Armonk, NY, USA). The significant level was considered as α = 0.05.

## Results

### Conceptual and item equivalence 

Following the literature review, the expert panel appraised the concept of toothache related behaviors in Iran and the original DDQ. It was found that DDQ concepts were valid in Iranian culture; however, an item was added to the first section of DDQ, which asked if anyone else except parents, such as a nurse or kindergarten teacher, had noticed the pain or not, regarding to Iranian cultural context.

One of the members of the panel rated the two items “earache when chewing” and “earache during the day” as less relevant (Table 1). Finally, the panel members validated all 12 items of the original DDQ.

**Table 1:** Expert panel’s evaluation of DDQ items’ relevancy regarding to Iranian cultural context

DDQ items	Scores*
1) Use their back teeth instead of their front teeth to bite things?	3,3,4,4
2) Stop eating sweets shortly after starting to eat them?	4,4,4,4
3) Start crying while eating a meal?	4,4,4,4
4) Have difficulty brushing the upper teeth?	4,3,3,3
5) Have difficulty brushing the lower teeth?	4,3,3,3
6) Complain of an earache while eating a meal?	4,2,4,4
7) Complain of an earache during the day?	3,2,3,4
8) Complain of an earache at night?	4,3,3,4
9) Have any difficulty in chewing?	4,4,4,4
10) Use only the teeth on one side for chewing?	3,4,4,4
11) Suddenly grab their cheek while eating?	4,4,4,4
12) Suddenly cry at night?	3,4,3,4

* Items relevancy based on expert panel’s evaluation; the panel members chose one of the following 4-point Likert scale: 1 not relevant, 2 little relevance, 3 relevant with minor modifications, 4 relevant.

The English-Persian translations and reverse translations were reviewed by the expert panel members and then, the back-translated consensus version was sent to the author of the original questionnaire (Table 2). The author confirmed the final; however, a minor revision was added and the term “objects” became replaced with “things” in the first question.

**Table 2:** The items of the original version and the adapted Persian version of the Dental Discomfort Questionnaire (P-DDQ)

Original DDQ	Adapted P- DDQ
Section 1: Toothache items	Section 1: Toothache items
1. Does your child have toothache? If sometimes or often is the:	1) Does your child have a toothache? If sometimes or often is the:
a. Toothache during meals	2) Does your child have a toothache during meals?
b. Toothache during day	3) Does your child have a toothache during the day?
c. Toothache during the night	4) Does your child have a toothache during the night?
2 a. Do you notice the toothache yourself?	5) Did you notice your child’s toothache yourself?
b. Does your child indicate the toothache to you?	6) Did your child report their toothache to you?
	7) Did anybody else (e.g., kindergarten trainer or babysitter) notice your child’s toothache?
Section 2: Oral habits items	Section 2: Oral habits items
Is your child:	Does your child:
1. Biting things off with their back teeth instead of their front teeth?	1) Use their back teeth instead of their front teeth to bite things?
2. Putting sweets away just after starting eating?	2) Stop eating sweets shortly after starting to eat them?
3. Starting to cry during meals?	3) Start crying while eating a meal?
4. a. Having problems with brushing upper teeth?	4) Have difficulty brushing the upper teeth?
b. Having problems with brushing lower teeth?	5) Have difficulty brushing the lower teeth?
5. a. Complaining about earache during eating?	6) Complain of an earache while eating a meal?
b. Complaining about earache during the day?	7) Complain of an earache during the day?
c. Complaining about earache at night?	8) Complain of an earache at night?
6. Having problems chewing?	9) Have any difficulty in chewing?
7. Chewing at one side?	10) Use only the teeth on one side for chewing?
8. Suddenly grabbing his/her cheek during eating?	11) Suddenly grab their cheek while eating?
9. Suddenly crying at night?	12) Suddenly cry at night?

### Operational equivalence

In order to test DDQ in the target population, 15 parents were invited to be interviewed. Most of the participants could not comprehend the examples in the DDQ introduction. Therefore, the section was substituted with two following alternative sentences: "Please fill out the questionnaire considering your child’s oral habits and behaviors” and “Mark the best choice using the symbol (√) for each question”.

Feedbacks of the interviews revealed that the Arabic numbers were used instead of a, b, c, 2a, 2b alphabets in order to separate question in part I (Toothache) and avoid the confusion of participants. In addition, the toothache and oral habit sections were provided in two separated pages to prevent the possible confusions. In the original version, the choices for each item were at the top of the page, which confused the respondents. To make the questionnaire more understandable, the choices "never", "sometimes", and "repeatedly" were provided next to each item. Moreover, the following question was added to the toothache section in P-DDQ: "Has anyone else noticed (nurse, kindergarten teacher or ...) the child's toothache?"

### Testing internal consistency and reliability

An acceptable level of internal consistency was observed between DDQ items (Cronbach’s alpha= 0.769). Cronbach’s alpha did not increase after removal of each of the 12 items. All of the items showed a moderate (kw&gt;0.60) or high (kw&gt; 0.80) level of agreement except two items of “earache during night”(kw=0) and “suddenly crying at night”(kw=0.512) (Table 3). The two items with small kw were not excluded from the questionnaire because there was not any increase in Cronbach’s alpha after their removal.

**Table3:** Results of Varimax rotation Factor Analysis and test-retest reliability for DDQ Items

DDQ items	Factor 1 “Ear problems”	Factor 2 “Function”	Factor 3 ”Difficulty in brushing teeth”	Weighted kappa coefficient (standard error)
Earache during eating	0.954	0.169	-0.051	1(0.00)
Earache during the day	0.768	-0.030	0.131	1(0.00)
Earache during the night	0.808	0.087	0.074	0.00
Putting sweets away after starting eating	0.443	0.585	-0.113	0.634 (0.170)
Chewing problem	0.102	0.662	0.417	0.916 (0.080)
Chewing at one side	-0.167	0.766	0.012	0.741 (0.128)
Grabbing cheek during eating	0.203	0.652	0.280	0.740 (0.136)
Crying at night	0.105	0.590	-0.055	0.512 (0.112)
*Biting with molars instead of anterior teeth	0.089	-0.051	0.297	0.675 (0.182)
*Crying while eating	-0.048	0.383	0.416	0.917 (0.080)
Difficulty in brushing upper teeth	-0.021	0.141	0.874	0.750 (0.165)
Difficulty in brushing lower teeth	-0.010	0.117	0.843	0.771 (0.142)
Eigenvalues	3.258	2.162	1.331
% of variance	27.150	18.021	11.094

*Factor loading less than 0.45

### Exploratory factor analysis

The appropriateness of the data for factor analysis was confirmed by the KMO index (KMO= 0.772); also, Bartlett’s test showed that the sphericity assumption was held (p< 0.001). EFA demonstrated that almost all the 12 items of the questionnaire were involved in three factors (Eigenvalues>1) and the factor loadings of each item were in accordance with Table 3. The mentioned three factors were accounted for approximately 56% of the total variance. 

Since the first factor included earache items, it was termed "ear problems". The second factor included the items that were related to chewing and sleeping problems and was termed as “functional domain”. The third factor was “difficulty in brushing teeth” and included difficulty in brushing upper and lower teeth items. The items “using the molar teeth instead of the front teeth” and “crying while eating” had a factor loading less than 0.45; therefore, they were not embedded in any of the factors.

### Construct and discriminant validity

Twelve parents (20%) stated that their children had never experienced toothache and 48 parents (80%) reported the toothache (sometimes or often). As it was shown in Table 4, children with parent-reported toothache had a higher DDQ score (median=7, IQR=14) compared to children without parent-reported toothache (median=0, IQR=4) (*p*< 0.001).

**Table 4:** The total scores of Persian version of Dental Discomfort Questionnaire (P-DDQ) in different clinical groups

Group	N	Total P-DDQ score		p
		Median	IQR	
Children with parent-reported toothache	48	7	14	<0.001*
Children with no parent-reported toothache	12	0	4	
children with toothache and decayed teeth	48	7_A_	14
children with decayed teeth without toothache	6	1.5_B_	4	<0.001†
children without toothache and decayed teeth	6	0_B_	0	

*: IQR: Interquartile Range

**: Mann-Whitney U test

†: Kruskal-Wallis H test

Median values with different capital Letters were statistically significant (Dunn’s Post-hoc test)

There was a positive relationship between the numbers of decayed teeth and DDQ scores (Spearman’s r= 0.47, p< 0.001). Moreover, a significant correlation was observed between DDQ scores and dmft (Spearman’s r= 0.439, p< 0.0001). In other words, the higher the numbers of decayed teeth and dmft led to the increase of DDQ score.

Considering the discriminant validity, Kruskal-Wallis H test revealed that there was a significant difference between DDQ score in the three clinical groups (p< 0.001). Dunn's post-hoc test showed that DDQ scores in group 1 (median=7) was significantly higher than in group 2 (Median=1.5 and 0, respectively) (both p< 0.001). However, there was not any significant difference between groups 2 and 3 (p> 0.05) (Table 4).

## Discussion

P-DDQ cross-cultural adaptation and validation procedures were conducted in current study. P-DDQ was a valid and reliable tool to assess the dental pain and discomfort among young Iranian children.

For conceptual equivalence, the expert panel found that DDQ concepts were valid in Iranian culture. They only added an item to the first section of DDQ. The panel validated all 12 items of the second section of DDQ for item equivalence. In addition, semantic equivalence was performed and the author of the original DDQ confirmed the final version after a minor revision. Following a pre-test in target population, necessary changes based on the participants’ feedback were made to the P-DDQ. Finally, P-DDQ became cross-culturally adapted in order to be applied in Iran.

Internal consistency was acceptable between the items of DDQ (Cronbach’s alpha= 0.769).Test-retest reliability showed moderate and high levels of agreement for DDQ items except for two items of “earache during night” and “crying at night”. However, there was not any increase in Cronbach’s alpha after removal of these two items; therefore, they were kept in P-DDQ. Because children may complain about experiencing pain at night because of various reasons, it seemed that parents were confused with the item that was related to the night earache complaints of their children. Versloot et al. [ 7 ] removed all three items related to earache in the short form of DDQ. Thereafter, the item should be considered doubtfully in further studies.

EFA showed that except two items, almost all 12 items of the questionnaire were involved in three factors. Construct and discriminant validity was also confirmed for the cross-culturally adapted version.

In a similar study, three earache items were removed from the questionnaire because it was found that earache could not be considered as s toothache-related behavior [ 7 ]. However, in current study, it was revealed that only one of the members of the expert panel rated two earache items as less relevant. As some children spend most of their time in kindergartens or with caregivers, the expert panel added a question to the first part of P-DDQ, which asked that if anyone else except parents, such as a nurse or kindergarten teacher, had noticed the pain or not.

The term “your child” from the original questionnaire was replaced with “child” in the Brazilian version [ 17 ]. In the Persian version, the term “your child” was applied, which was similar to the original version, because children were referred to the dental clinic with their parents.

Similar to the Brazilian study [ 17 ], the examples provided in DDQ introduction were substituted with two alternative sentences. According to the feedbacks of interviews, several modifications were made in order to achieve a better perception of Iranian participants.

A previous study showed that DDQ may be a uni-dimensional scale [ 7 ]. EFA revealed that almost all adapted 12 items were included into three independent factors except two items of “bite with molars instead of anterior teeth” and “crying while eating”. The first factor included earache problems-related items, the second one was about brushing problems, and the last factor included most of the items that assessed sleeping and chewing difficulties. Two above-mentioned items that did not fit into any factors were inconsistent with the previous Brazilian study [ 11 ]. These two items were included in sleeping and chewing factor in the mentioned study [ 11 ]. This could be due to the variations in pain-related behaviors and cultures of different countries. Findings derived from the interviews revealed that most of the parents did not pay attention to the mode of biting in their children. According to the Brazilian version of DDQ, this item was not significantly different among children with or without toothache, and it was suggested that this issue be considered for future researches [ 11 ].

As observed in the similar Brazilian version [ 11 ], children with parent-reported toothache had a higher DDQ score compared to the children without any parent-reported toothache. Current study showed that DDQ scores in children with toothache and decayed teeth were significantly higher than those without toothache (with or without decayed teeth), which was in accordance with the investigation carried out by Bansal et al. [ 10 ].

In current research, all 12 items of the adopted questionnaire were evaluated; however, only 7 items were tested in the Brazilian study [ 11 ]. It seems that all 12 items could demonstrate the dental discomfort traits in children perfectly in P-DDQ. The authors of the original version of DDQ removed the three earache items, as well as “crying at night” item in their short form of DDQ in order to achieve a better reliability [ 7 ]. It is recommended that further investigations compare the short form with the original one with the purpose of achieving the best psychometric properties for the Persian version.

To our knowledge, very few similar studies were performed for cross-cultural adaptation of DDQ worldwide [ 11 - 17 ]. Lack of gold standard for toothache assessment in young children was an inherent shortage. Therefore, parents’ reports were the only solution. Although the sample size was sufficient for psychometric assessments, it was not large enough to generalize the results to the entire population of Iranian children. 

Further studies with larger sample sizes and other clinical indices, such as PUFA index which records the presence of severely decayed teeth with visible pulpal involvement (P/p), ulceration caused by dislocated tooth fragments (U/u), fistula (F/f) and abscess (A/a); have to be carried out in order to detect the toothache in young children using P-DDQ. Moreover, DDQ should be adapted in other countries with the purpose of assessing the validity and reliability in various cultures. Because children referred to dental school were evaluated in current study, the number of children without decayed teeth was small. It is suggested that future studies evaluate children from kindergartens or health centers instead of dental clinics; however, this evaluation could not be conducted in current study due to COVID-19 pandemic and cross-country lockdown.

To the best of our knowledge, this is the first study that assessed P-DDQ validity and reliability. Therefore, policy-makers and oral health planners could apply this version in further investigations to detect the toothache in epidemiological studies or in children with mental disabilities before the occurrence of sever dental caries-resulted clinical and life quality adverse effects in Iran.

## Conclusion

The cross-culturally adapted version of DDQ led to an acceptable level of reliability and validly. P-DDQ score had a significant correlation with dmft index as a clinical index and was statistically different between those with and without parental report of toothache. Furthermore, it was found that P-DDQ could be implemented as a reliable instrument for the early toothache detection in young Iranian children.

## Acknowledgement

The authors thank the Research Vice-Chancellor of Shiraz University of Medical Science for supporting this research (Grant# 21389). This manuscript is based on the thesis by Dr. Mohamadjavad Yousefimaaghoul.

## Conflict of Interest

The authors declare that they have no conflict of interest.

## References

[1] Von Baeyer CL, Uman LS, Chambers CT, Gouthro A (2011). Can we screen young children for their ability to provide accurate self-reports of pain?. Pain.

[2] Herr K, Coyne PJ, Ely E, Gélinas C, Manworren RCB ( 2019). Pain Assessment in the Patient Unable to Self-Report: Clinical Practice Recommendations in Support of the ASPMN 2019 Position Statement. Pain Manag Nurs.

[3] Boeira G, Correa M, Peres K, Peres M, Santos I, Matijasevich A, et al ( 2012). Caries is the main cause for dental pain in childhood: findings from a birth cohort. Caries Res.

[4] Barasuol JC, Santos PS, Moccelini BS, Magno MB, Bolan M, Martins Júnior PA, et al ( 2020). Association between dental pain and oral health‐related quality of life in children and adolescents: A systematic review and meta‐analysis. Community Dent Oral Epidemiol.

[5] Rauber ED, Menegazzo GR, Knorst JK, Bolsson GB, Ardenghi TM ( 2021). Pathways between toothache and children's oral health‐related quality of life. Int J Paediatr Dent.

[6] Cascella M, Bimonte S, Saettini F, Muzio MR ( 2019). The challenge of pain assessment in children with cognitive disabilities: Features and clinical applicability of different observational tools. J Pediatr Child Health.

[7] Versloot J, Veerkamp JSJ, Hoogstraten J ( 2006). Dental Discomfort Questionnaire: assessment of dental discomfort and/or pain in very young children. Community Dent Oral Epidemiol.

[8] Levine RS, Nugent ZJ, Pitts NB ( 2003). Pain prediction for preventive non-operative management of dentinal caries in primary teeth in general dental practice. Br Dent J.

[9] Rosli MNA, Ghani MA, Supaat S, Kharuddin AF, Ardini YD ( 2018). Dental discomfort questionnaire as an assessment tool in detecting early childhood caries. J Int Dent Med Res.

[10] Bansal P, Sujlana A, Pannu P, Kour R ( 2017). Dental Discomfort Questionnaire: correlated with clinical manifestations of advanced dental caries in young children. J Dent Specialities.

[11] Daher A, Versloot J, Leles CR, Costa LR ( 2014). Screening preschool children with toothache: validation of the Brazilian version of the Dental Discomfort Questionnaire. Health Qual Life Outcomes.

[12] Felipak PK, Menoncin BLV, Reyes MRT, Costa LR, de Souza JF, Menezes JVNB ( 2020). Determinants of parental report of dental pain and discomfort in preschool children- The Dental Discomfort Questionnaire. Int J Paediatr Dent.

[13] Penmetsa C, Penmetcha S, Cheruku SR, Mallineni SK, Patil AK, Namineni S ( 2019). Role of dental discomfort questionnaire-based approach in recognition of symptomatic expressions due to dental pain in children with autism spectrum disorders. Contemp Clin Dent.

[14] Alzahrani  AAH ( 2019). Parent perspectives on perceived dental pain and dental caries in Saudi schoolchildren with intel lectual disability. Spec Care Dent.

[15] Senirkentli GB, Tirali RE, Bani M ( 2021). Assessment of dental pain in children with intellectual disability using the den-tal discomfort questionnaire. J Intellect Disabil.

[16] Versloot J, Veerkamp JSJ, Hoogstraten J ( 2005). Dental discom-fort questionnaire for young children before and after treatment. Acta Odontol Scand.

[17] Daher A, Versloot J, Costa LR ( 2014). The cross-cultural pro-cess of adapting observational tools for pediatric pain as-sessment: the case of the Dental Discomfort Question-naire. BMC Res Notes.

[18] Aillón  IEV, Tello G, Corrêa-Faria P, Abanto J, Oliveira LB, Bönecker M ( 2020). Dental Pain in Preschool Children Us-ing the Brazilian Dental Discomfort Questionnaire and its Association with Dental Caries and Socioeconomic Fac-tors. Pediatr Dent.

[19] Kaviani N, Mohammadi GM, Mohammadi A, Ahmady N ( 2018). Evaluation of Dental Discomfort Questionnaire (DDQ) For Young Children Before and After Dental Treatment under General Anesthesia. J Biochem Tech.

[20] Toutouni H, Nokhostin MR, Amaechi BT, Zafarmand AH ( 2015). The prevalence of early childhood caries among 24 to 36 months old children of Iran: using the novel ICDAS-II method. J Dent.

